# Supplementary figures and images for: Genome-wide transcriptomics identifies an early preclinical signature of prion infection
Source: PLoS Pathog. 2020 Jun 29;16(6):e1008653. doi: 10.1371/journal.ppat.1008653 (PMC7360066; doi:10.1371/journal.ppat.1008653)

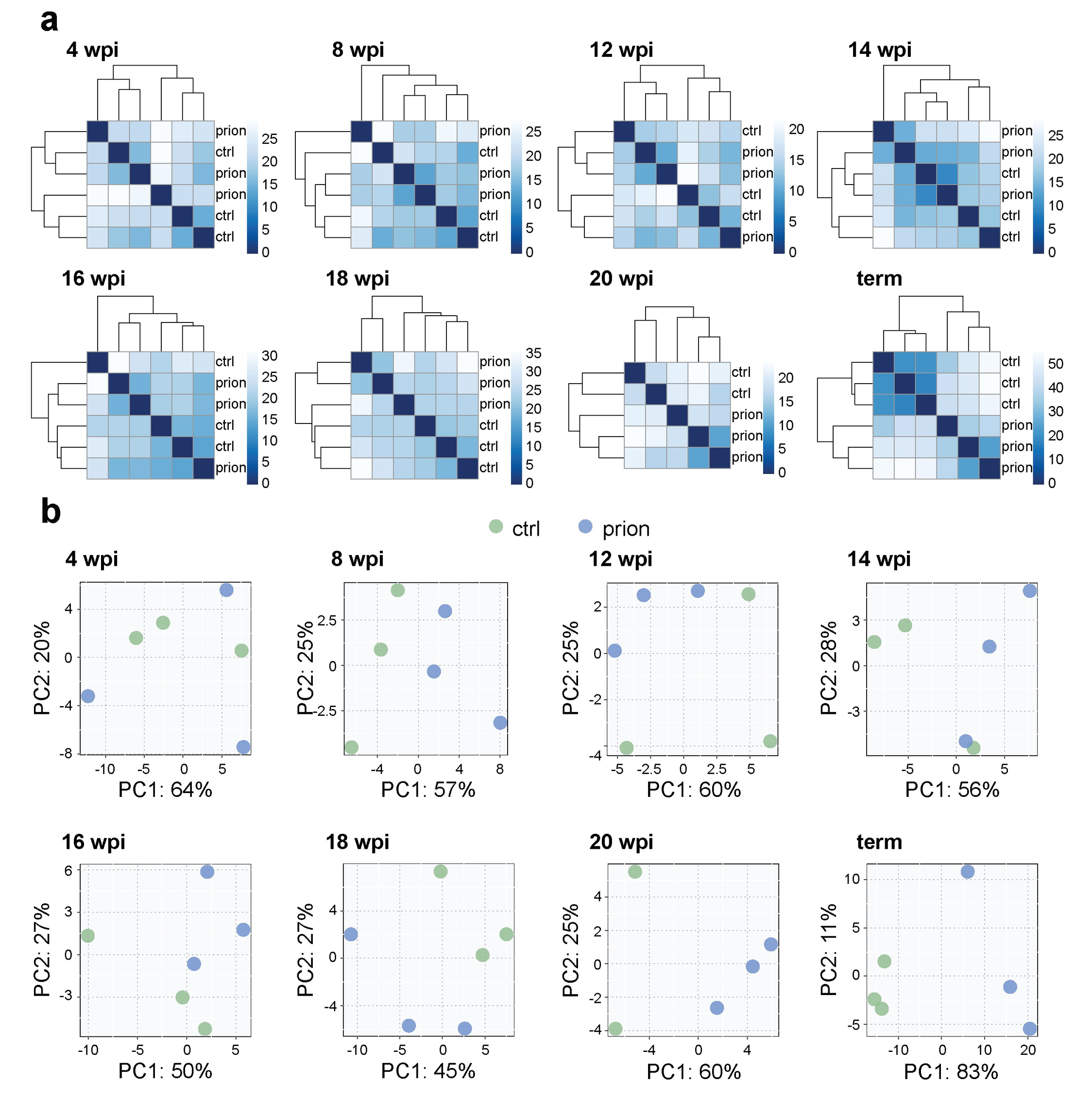

Supplement: S1 Fig — a, Hierarchical clustering based on Euclidean distances. Heatmaps depicting the sample distances at each time point based on RNAseq expression data. Control and prion-injected samples cluster from 18 wpi onwards. b, Principal component analysis of RNAseq samples at different time points revealing a separation of control (green) and prion-injected (blue) samples at 8 wpi, 20 wpi and the terminal stage. (TIF) [file ppat.1008653.s001.tif]

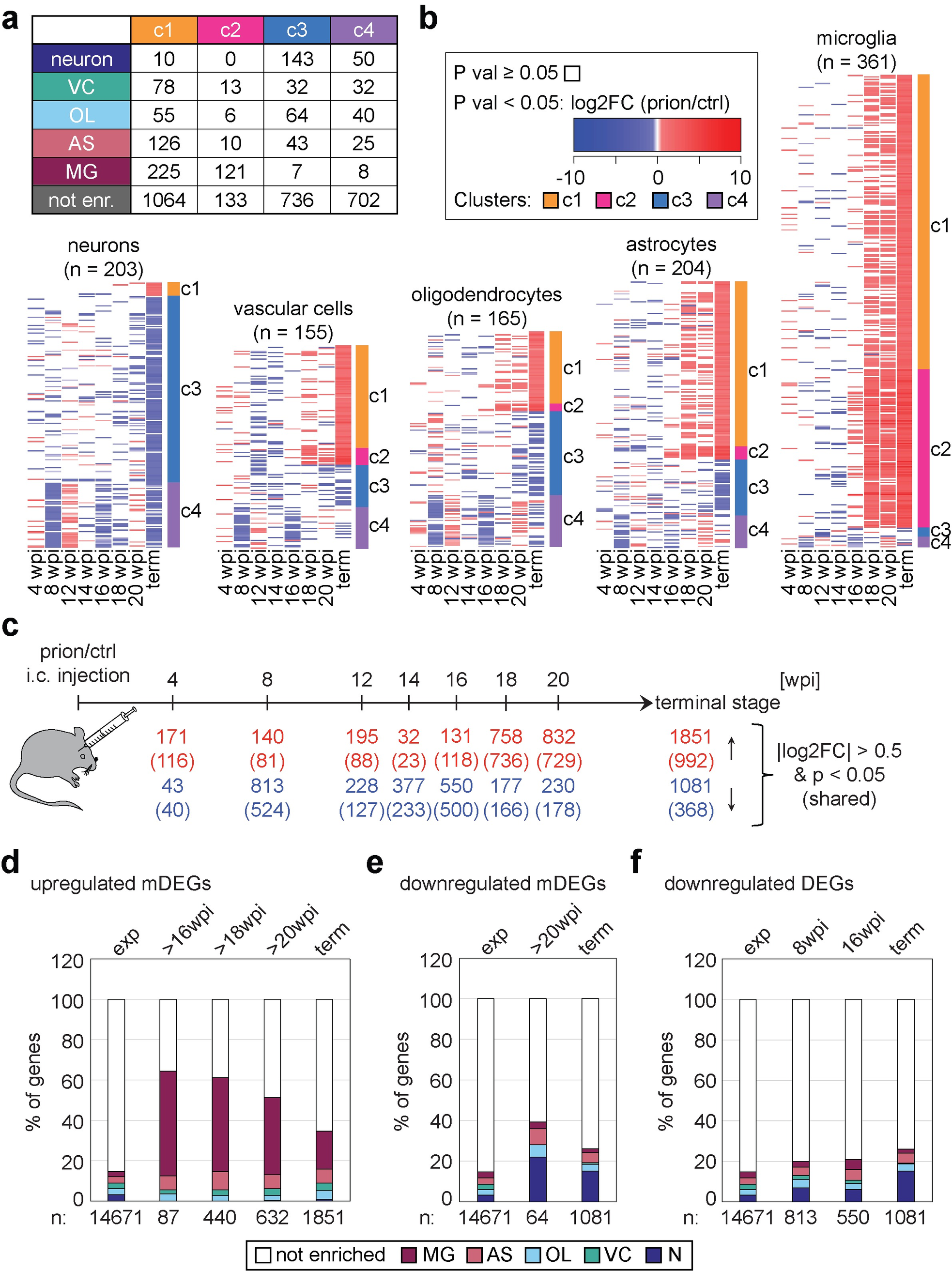

Supplement: S2 Fig — a, Table displaying the number of genes enriched in the different cell types and which cluster they belong to. b, Heat maps depicting the log2FC of DEGs known to be enriched in different cell types (only log2FC with p < 0.05 are colored). The corresponding clusters are indicated as a side bar. The height of the heatmap corresponds to the number of genes. c, Schematic depicting time points of prion inoculations. Number of upregulated and downregulated DEGs (|log2FC| > 0.5 and p < 0.05), and numbers of shared genes (in brackets) are indicated. d-f, Percentages of cell-type enriched genes within upregulated mDEGs (c), downregulated mDEGs (d) and downregulated genes at different time points (e). (TIF) [file ppat.1008653.s002.tif]

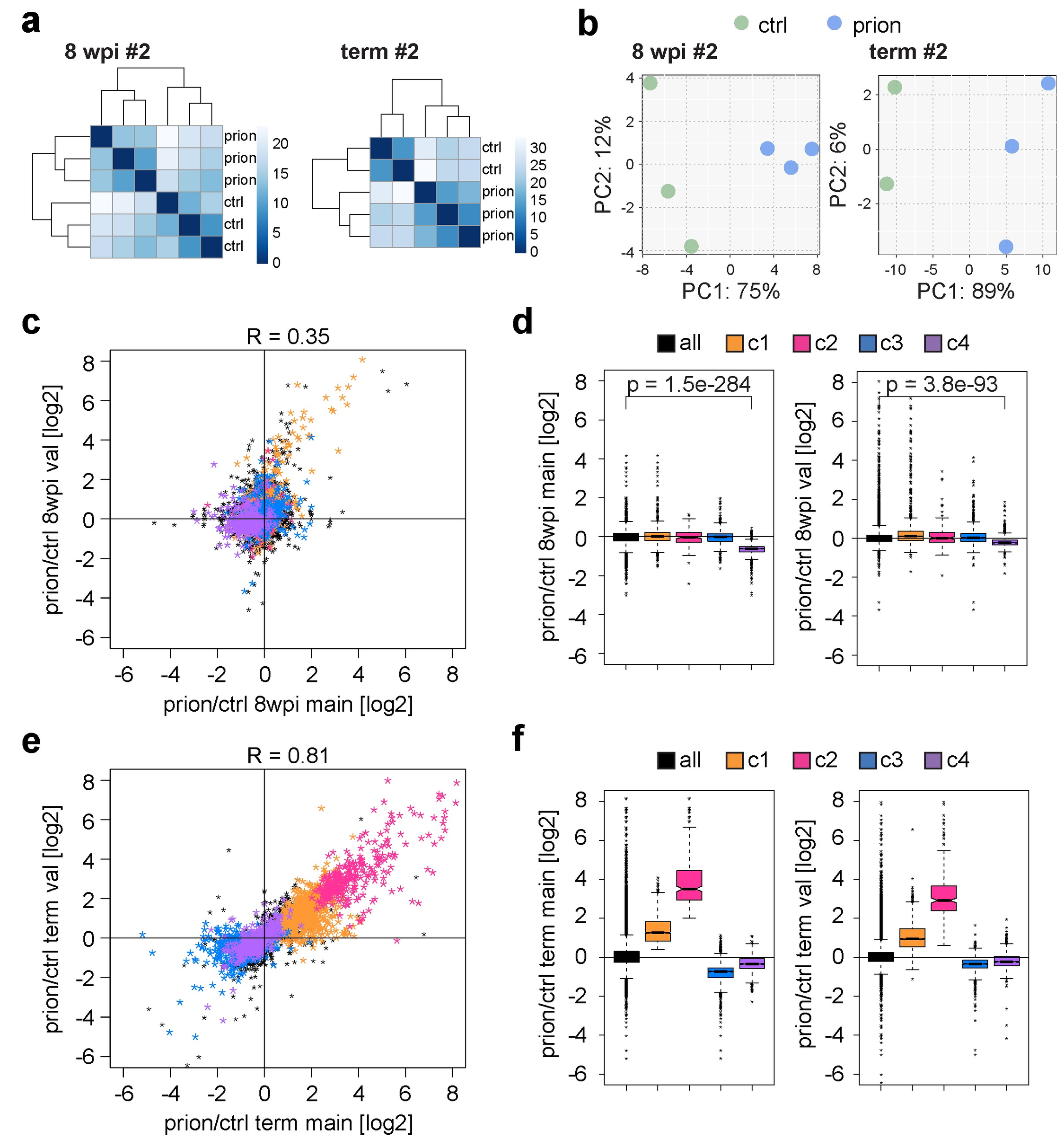

Supplement: S3 Fig — a, Hierarchical clustering based on Euclidean distances. Heatmaps depicting the sample distances based on RNAseq expression data. Control and prion-injected samples cluster at 8 wpi, and the terminal stage. b, Principal component analysis of RNAseq samples revealing a separation of control (green) and prion-injected (blue) samples at 8 wpi, and the terminal stage. c, Scatter plot depicting expressed genes. The change in expression at 8 wpi correlates between the main and the validation datasets (R = 0.29). Genes belonging to the clusters identified in Fig 1 are colored. d, Boxplots representing the log2FC distribution in the main (left panel) and validation (right panel) datasets at 8 wpi. Only expressed genes are included. The distribution of all genes (black) and genes belonging to the clusters identified in Fig 1 are shown. The difference in the log2FC distribution between expressed genes and cluster 4 genes was assessed with a t test. e, Scatter plot depicting expressed genes. The change in expression at the terminal stage highly correlates (R = 0.80) and genes belonging to the clusters identified in Fig 1 are colored. f, Boxplots representing the log2FC distribution in the main (left panel) and validation (right panel) datasets at the terminal stage. Only expressed genes are included. The distribution of all genes (black) and genes belonging to the clusters identified in Fig 1 are shown. The difference in the log2FC distribution between expressed genes and cluster 4 genes was assessed with a t test. (TIF) [file ppat.1008653.s003.tif]

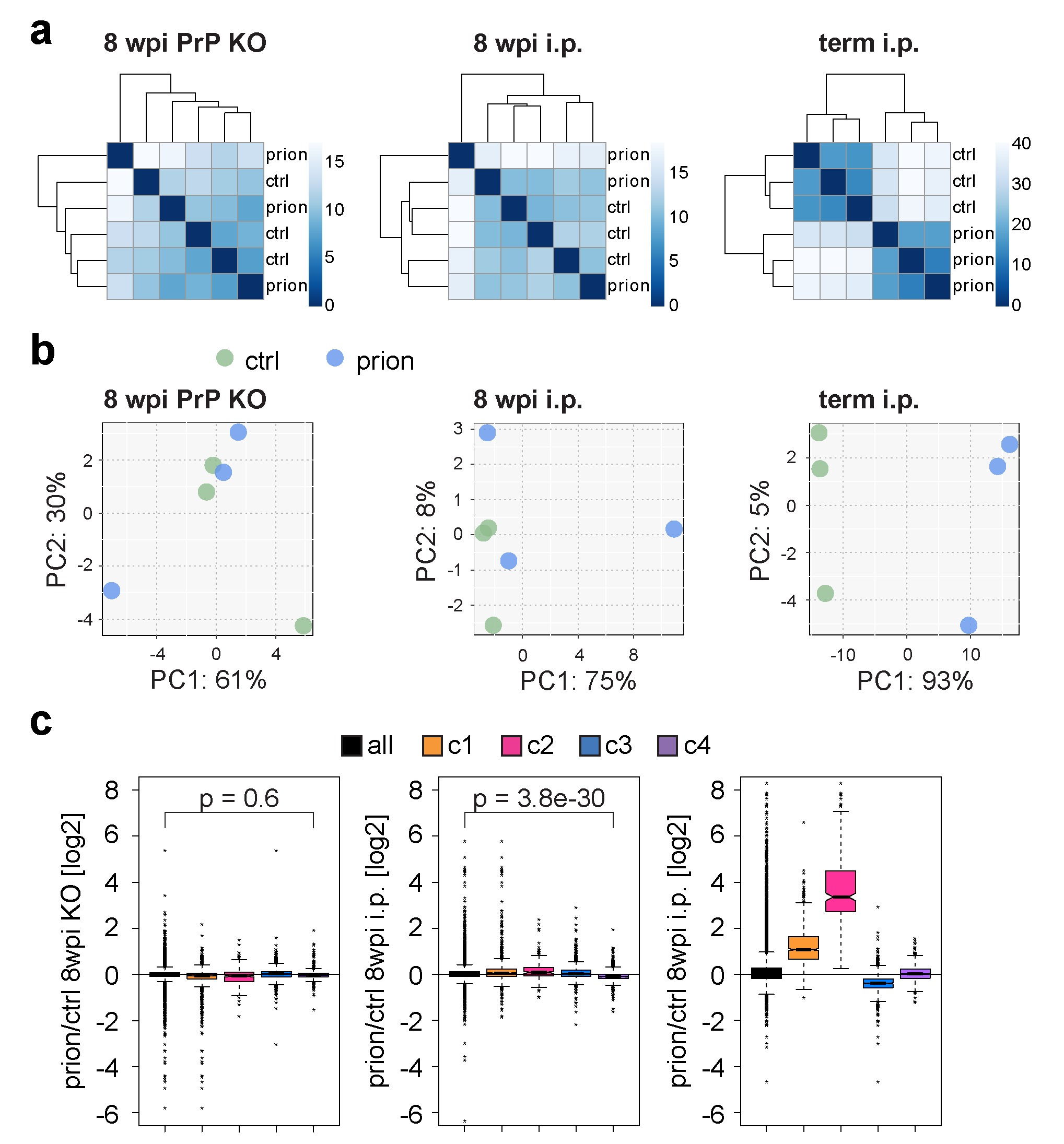

Supplement: S4 Fig — a, Hierarchical clustering based on Euclidean distances. Heatmaps depicting the sample distances based on RNAseq expression data. Control and prion-injected samples cluster at the terminal stage in intraperitoneally (i.p.) inoculated wild-type mice but not in intracerebrally (i.c.) inoculated PrP knockout (KO) mice, nor in intraperitoneally inoculated wild-type mice at 8 wpi. b, Principal component analysis of RNAseq samples revealing a separation of control (green) and prion-injected (blue) samples of intraperitoneally inoculated wild-type mice at the terminal stage. c, Boxplots representing the log2FC distribution of intracerebrally inoculated PrP knockout mice at 8 wpi and of intraperitoneally inoculated wild-type mice at 8 wpi and the terminal stage. Only expressed genes are included. The distribution of all genes (black) and genes belonging to the clusters identified in Fig 1 are shown. The difference in the log2FC distribution between expressed genes and cluster 4 genes was assessed with a t test. (TIF) [file ppat.1008653.s004.tif]

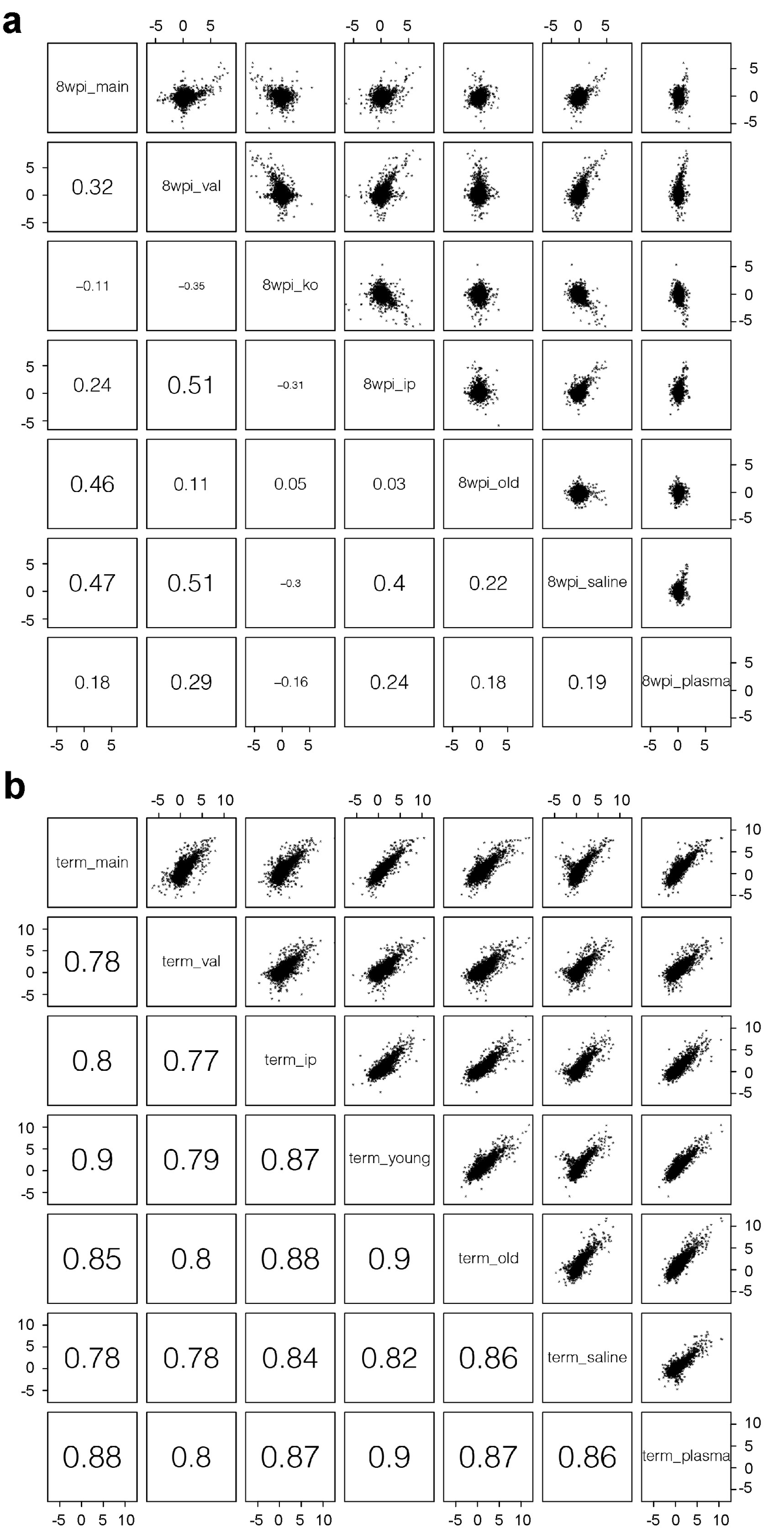

Supplement: S5 Fig — a, Cross-correlation plot of all 8 wpi comparisons included in this manuscript (main, validation, PrP knockout (ko), intraperitoneally (ip) inoculated, aged, saline-treated, plasma-treated). Shown are scatter plots comparing the 8 wpi log2FC between the groups and the corresponding R values (scaled according to R value). The PrP knockout samples do not correlate with any other sample. Among the others, the plasma-treated sample correlates least with the other 8 wpi samples. Only expressed genes are included. b, Cross-correlation plot of all terminal comparisons included in this manuscript (main, validation, intraperitoneally (ip) inoculated, young, aged, saline-treated, plasma-treated). Shown are scatter plots comparing the terminal log2FC between the groups and the corresponding R values (scaled according to R value). All samples strongly correlate with each other. Only expressed genes are included. (TIF) [file ppat.1008653.s005.tif]

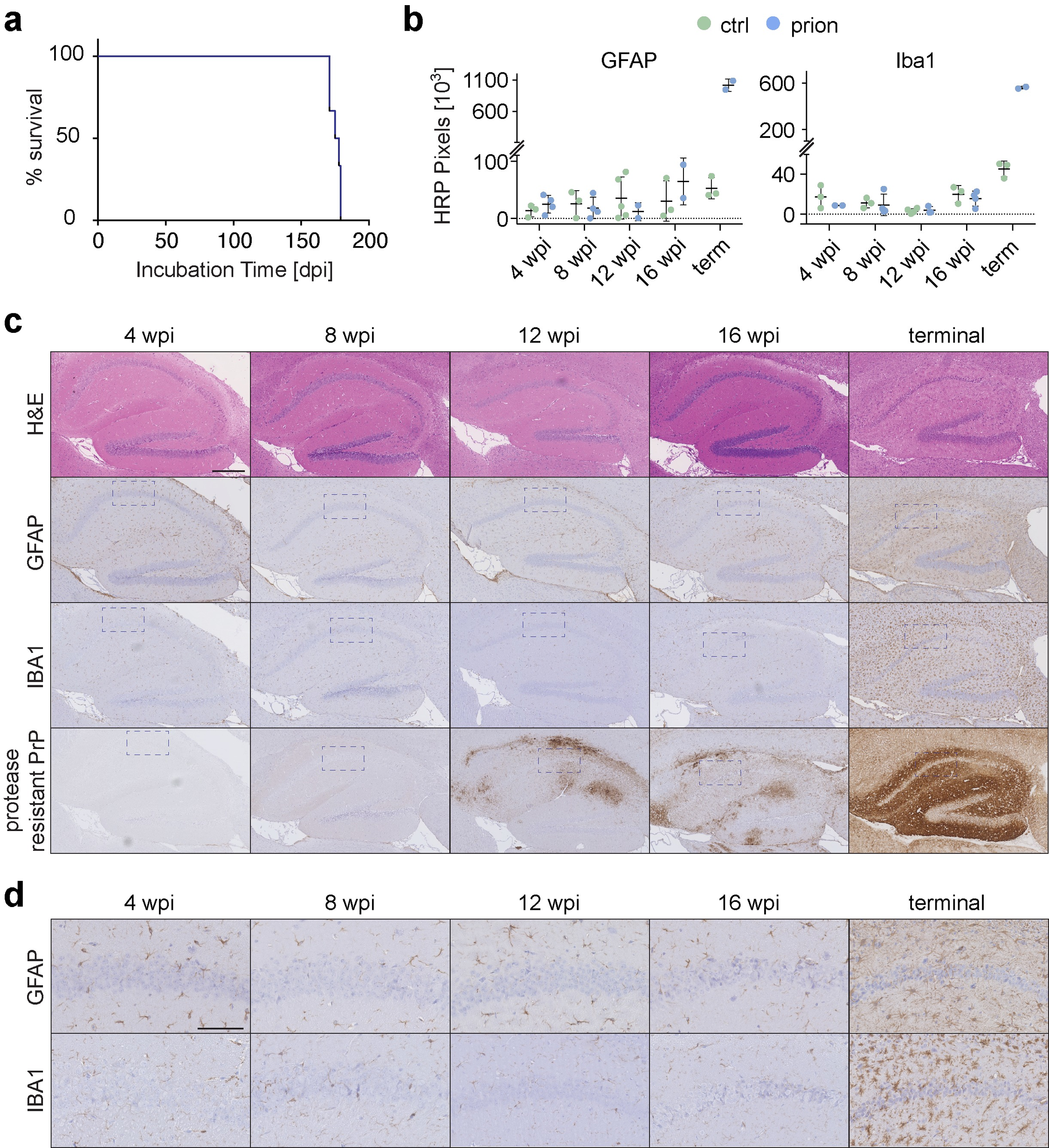

Supplement: S6 Fig — a, Survival curve of mice sacrificed at the terminal stage. Shown is % survival compared to days post inoculation (dpi). b, Quantification of high magnification images (including representative images shown in d). Error bars indicate standard deviations. c, Prion-inoculated mice were sacrificed at the indicated time points during disease progression. Brain section were stained with hematoxylin and eosin (H&E), GFAP (astrocyte marker), IBA1 (microglia marker) and SAF84 (detects only PrPSc after protease treatment). Shown are representative pictures at each timepoint. Rectangles correspond to approximate location of higher-magnification images shown in d. Scale bar in upper left panel: 250μm (applicable to all panels). d, Higher-magnification images of GFAP, IBA1 and SAF84 stainings shown in c. Scale bar in upper left panel: 100μm (applicable to all panels). (TIF) [file ppat.1008653.s006.tif]

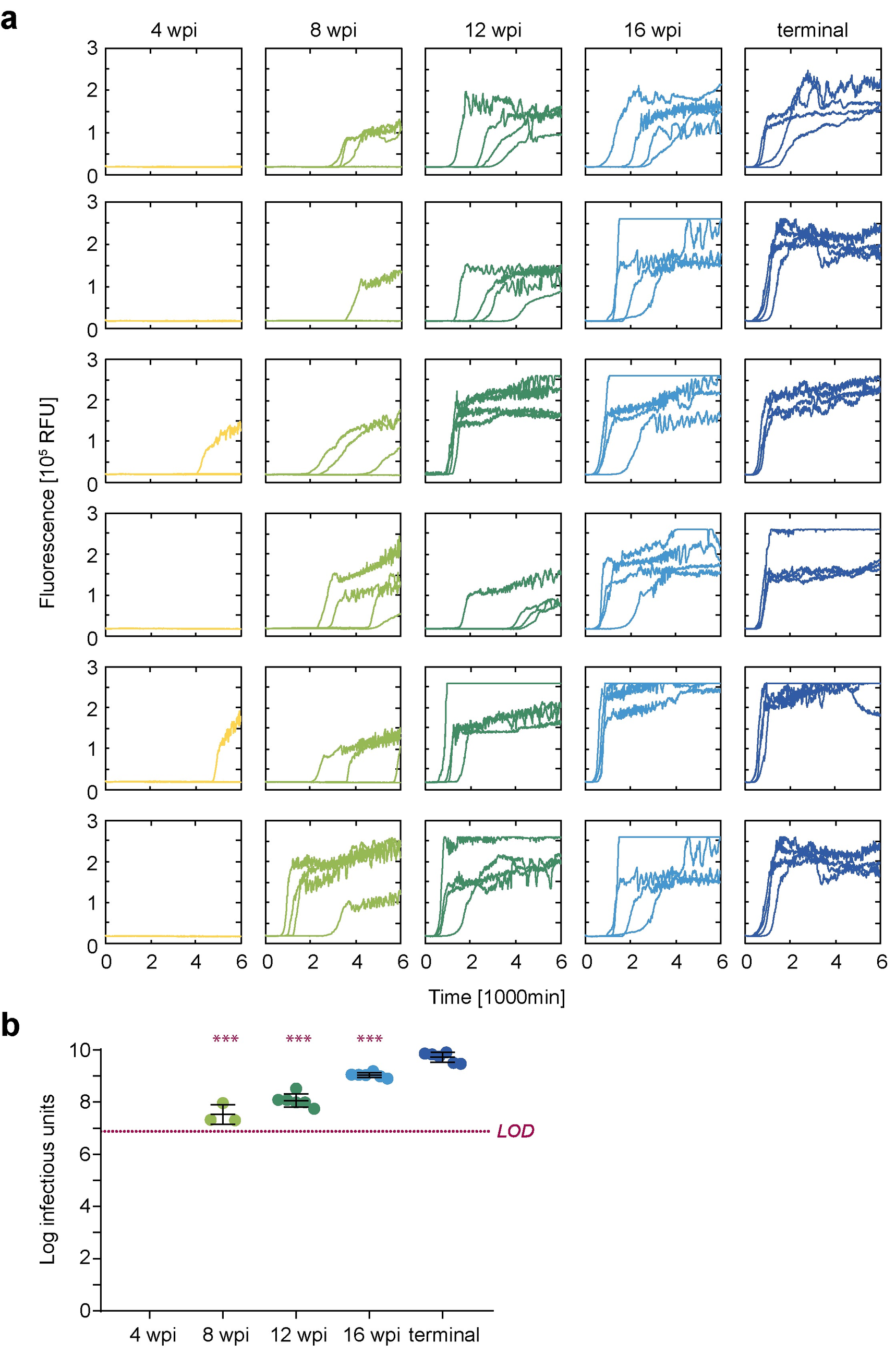

Supplement: S7 Fig — a, RT-QuIC reactions using brain homogenates of mice inoculated with prions and sacrificed at indicated time points. Each sample was tested in quadruplicates, and each plot corresponds to one mouse (n = 6). RFU: relative fluorescence units. b, Dot plot graph showing infectious units measured by standard scrapie cell assay (SSCA). Each dot represents one mouse, bars indicate standard deviations. 6 (out of 6) samples at 4wpi and 3 (out of 6) samples at 8 wpi were below the limit of detection (LOD). P values were calculated with a one-way ANOVA followed by Tukey’s multiple comparison test (***p<0.001; compared to terminal). (TIF) [file ppat.1008653.s007.tif]

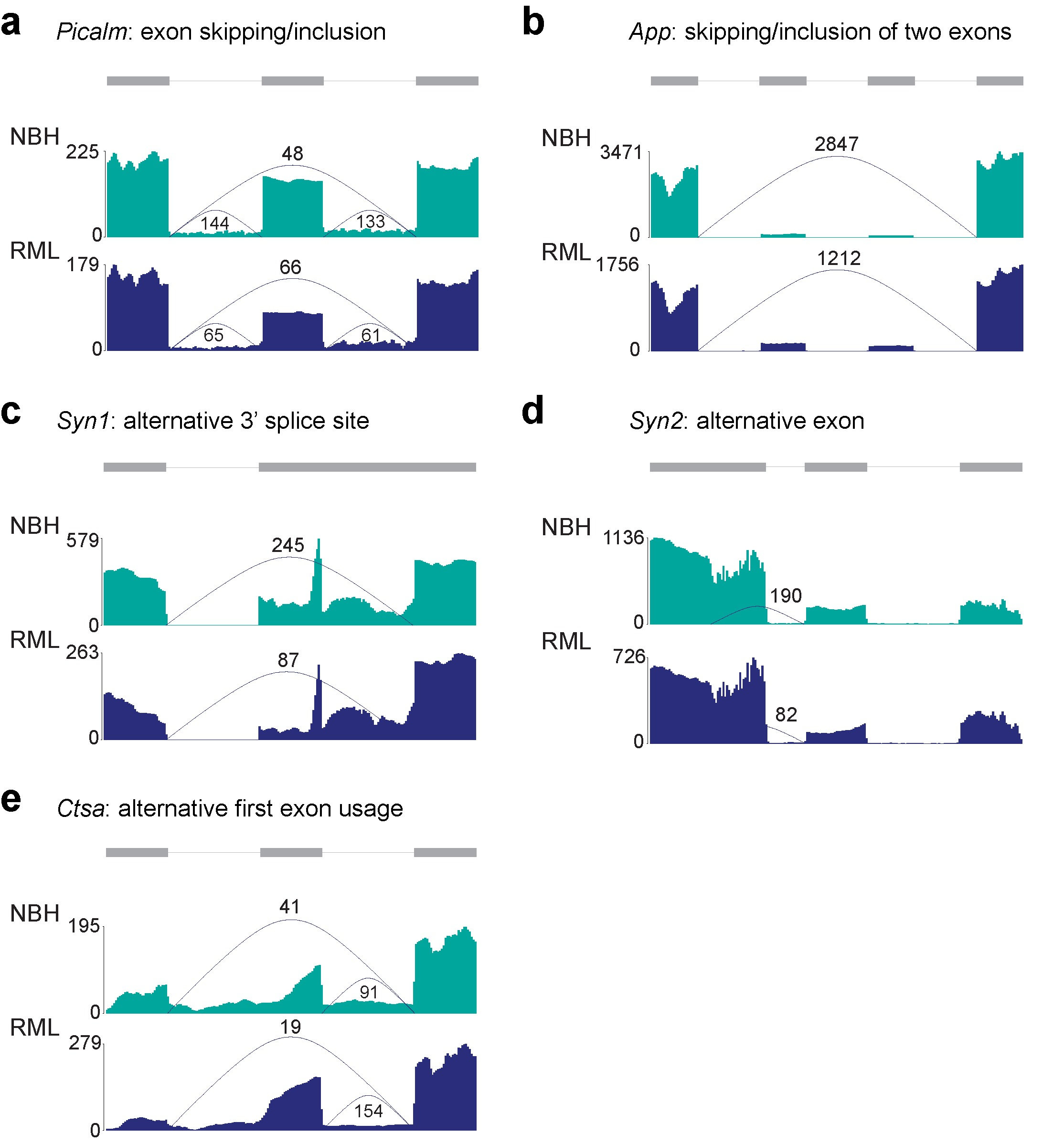

Supplement: S8 Fig — (Alternative) exons are shown in grey. Exons and introns are not drawn to scale. Average per-base exon read coverage and junction counts normalized to total read counts in control and prion diseased mice at the terminal time point are shown. Splicing events in Picalm (a), App (b), Syn1 (c), Syn2 (d), and Ctsa (e) were visualized with the plotSpliceGraph function of the SGSeq package in R. Shown events are indicated in S8 Fig (TIF) [file ppat.1008653.s008.tif]

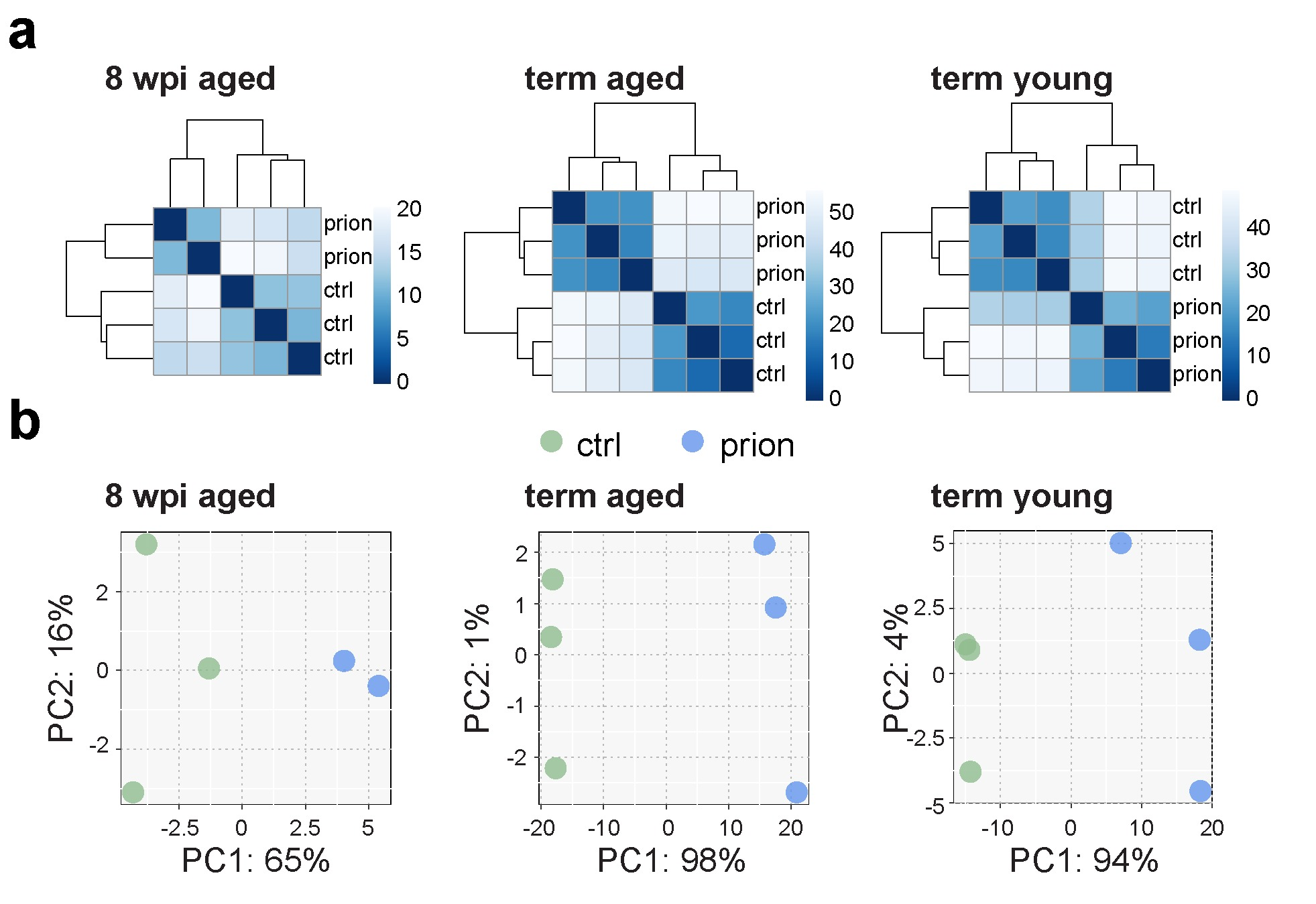

Supplement: S9 Fig — a, Hierarchical clustering based on Euclidean distances. Heatmaps depicting the sample distances based on RNAseq expression data. Control and prion-injected samples cluster at 8 wpi and the terminal stage in aged mice, and at the terminal stage in young mice. b, Principal component analysis of RNAseq samples revealing a separation of control (green) and prion-injected (blue) samples at 8 wpi and the terminal stage in aged mice, and at the terminal stage in young mice. (TIF) [file ppat.1008653.s009.tif]

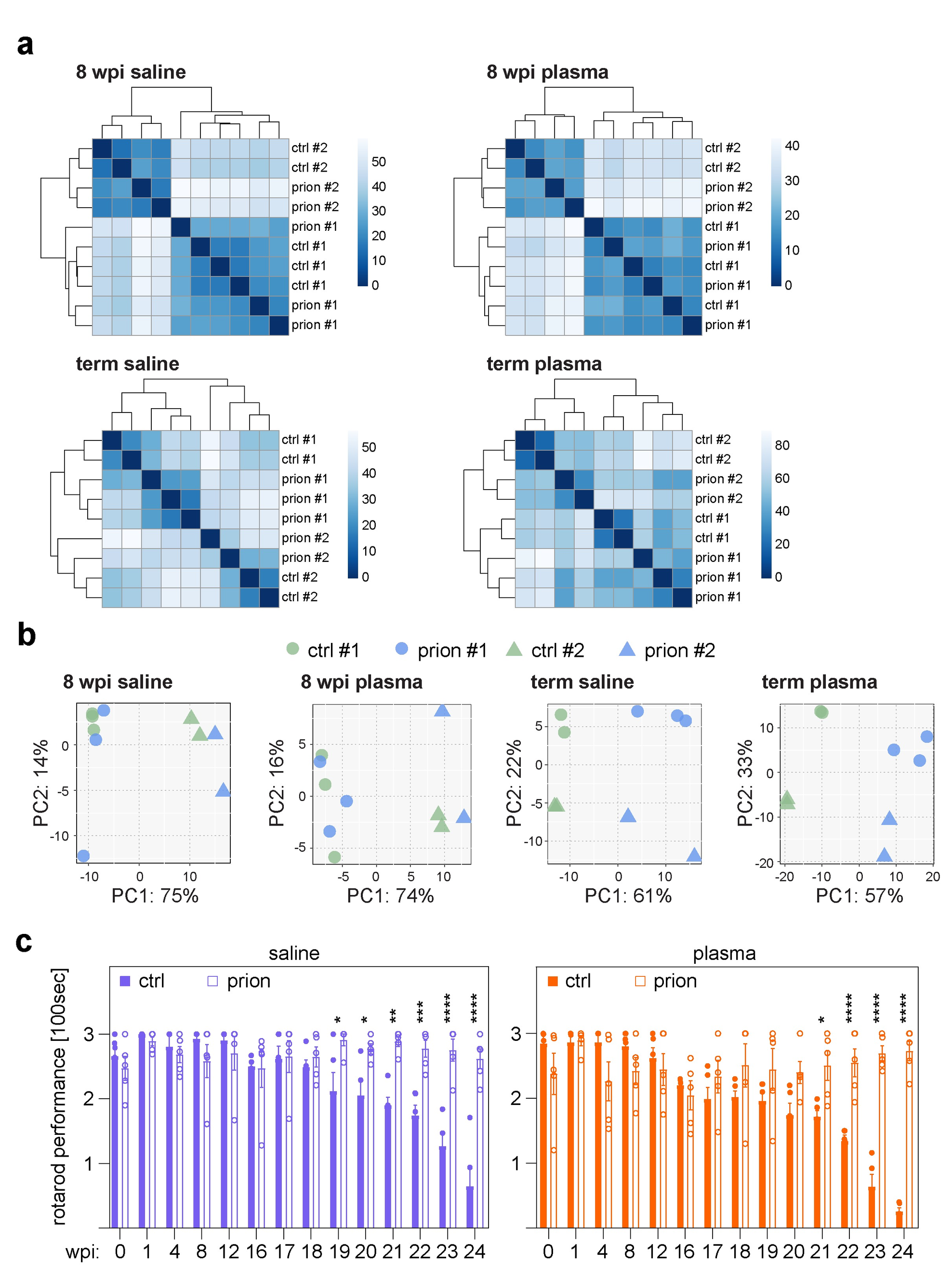

Supplement: S10 Fig — a, Hierarchical clustering based on Euclidean distances. Heatmaps depicting the sample distances based on RNAseq expression data. Samples cluster predominantly according to cohorts. b, Principal component analysis of RNAseq samples revealing a separation of control (green) and prion-injected (blue) samples at the terminal stage. Samples additionally separated according to the run of RNA isolation/processing/sequencing (run #1 versus run #2). The batch effect of the different run was accounted for during the analysis. c, Rotarod performance of saline (left panel) and plasma-treated (right panel) prion-inoculated mice at specified time points during disease progression (pre = pre-inoculation). Bar plots display the mean latency +/- SEM to fall in 100 seconds, with each dot representing one individual mouse. P values were calculated with a one-way ANOVA followed by Tukey’s multiple comparison test (*p<0.05; **p<0.01; ***p<0.001; ****p<0.0001; compared to 1 wpi). (TIF) [file ppat.1008653.s010.tif]
